# From bench to bedside – current clinical and translational challenges in fibula free flap reconstruction

**DOI:** 10.3389/fmed.2023.1246690

**Published:** 2023-10-11

**Authors:** Helena Baecher, Cosima C. Hoch, Samuel Knoedler, Bhagvat J. Maheta, Martin Kauke-Navarro, Ali-Farid Safi, Michael Alfertshofer, Leonard Knoedler

**Affiliations:** ^1^Department of Plastic, Hand and Reconstructive Surgery, University Hospital Regensburg, Regensburg, Germany; ^2^Medical Faculty, Friedrich Schiller University Jena, Jena, Germany; ^3^Division of Plastic Surgery, Department of Surgery, Yale New Haven Hospital, Yale School of Medicine, New Haven, CT, United States; ^4^Division of Plastic Surgery, Department of Surgery, Brigham and Women’s Hospital, Harvard Medical School, Boston, MA, United States; ^5^Department of Plastic Surgery and Hand Surgery, Klinikum rechts der Isar, Technical University of Munich, Munich, Germany; ^6^College of Medicine, California Northstate University, Elk Grove, CA, United States; ^7^Craniologicum, Center for Cranio-Maxillo-Facial Surgery, Bern, Switzerland; ^8^Faculty of Medicine, University of Bern, Bern, Switzerland; ^9^Division of Hand, Plastic and Aesthetic Surgery, Ludwig-Maximilians-University Munich, Munich, Germany

**Keywords:** fibula free flap, mandibular reconstruction, artificial intelligence, 3D printing, computer-aided design, CAM, bioprinting

## Abstract

Fibula free flaps (FFF) represent a working horse for different reconstructive scenarios in facial surgery. While FFF were initially established for mandible reconstruction, advancements in planning for microsurgical techniques have paved the way toward a broader spectrum of indications, including maxillary defects. Essential factors to improve patient outcomes following FFF include minimal donor site morbidity, adequate bone length, and dual blood supply. Yet, persisting clinical and translational challenges hamper the effectiveness of FFF. In the preoperative phase, virtual surgical planning and artificial intelligence tools carry untapped potential, while the intraoperative role of individualized surgical templates and bioprinted prostheses remains to be summarized. Further, the integration of novel flap monitoring technologies into postoperative patient management has been subject to translational and clinical research efforts. Overall, there is a paucity of studies condensing the body of knowledge on emerging technologies and techniques in FFF surgery. Herein, we aim to review current challenges and solution possibilities in FFF. This line of research may serve as a pocket guide on cutting-edge developments and facilitate future targeted research in FFF.

## 1. Introduction

Facial defects can be caused by neoplasms, infection, trauma, or congenital anomaly. Patients experience a variety of functional sequels such as malocclusion and loss of teeth, swallowing and speaking difficulties, and airway obstruction (1). Besides functional impairments, facial disfigurement *per se*, and the resulting social and psychological effects, represent an additional patient burden (2, 3).

Consequently, reconstruction of bony and soft tissue facial defects remains a persisting clinical challenge. Since its establishment in 1989, fibula free flap (FFF) became a reliable tool in the head and neck armamentarium representing the gold standard for the reconstruction of segmental defects of the mandible and maxilla (4). FFF unifies various clinical strengths such as low donor-site morbidity, bicortical bone structure, and reliable clinical outcomes. Currently, FFF is being used in over 45% of all mandible reconstruction cases, and has been gaining popularity (5, 6).

Typically, FFF is raised with a skin island, which enables composite-tissue reconstruction and visual flap monitoring at the same time (7). In the era of virtual surgical planning (VSP), artificial intelligence (AI), computer-aided design (CAD), computer-aided manufacturing (CAM), three-dimensional (3D) printing, and novel molecular investigations, the technique of FFF underwent a series of refinements. Consequently, each working step of FFF reconstruction, including preoperative diagnostics and procedure planning, intraoperative workflow optimization, and postoperative monitoring has been impacted as a result of advancements in the field. However, there is a paucity of studies condensing the latest advancements in FFF surgery. Herein, we aim to provide a panoramic overview of the most recent innovations in FFF reconstruction and summarize the most promising research findings. Ultimately, this line of research may help generate novel research ideas and guide the way toward translational trials and clinical use of cutting-edge technology for advancing FFF surgery.

## 2. Preoperative planning – how to optimize procedure planning?

### 2.1. Virtual surgical planning

In the early phase of FFF surgery, the fibula was shaped in freehand technique by the surgeon, using the patient’s contralateral anatomic site as a template in order to guide the osteotomies and plate placements. However, this freehand molding of the straight fibula bone into the 3D-curved mandible is challenging and failure in contouring the new mandible may cause malocclusion, mandible deformity, such as exorbitant gonial or mental projection, or torque on the condylar head. Further, mandibular dysfunction, as well as prosthetic failure can result from an unphysiological-shaped mandible. To overcome these hurdles the preplating technique was introduced. This method involves molding a reconstructive plate on the patient’s native mandible prior to bone resection, aiming to achieve an optimal mandibular shape (7–9). However, manual techniques of fibula modeling and positioning to achieve proper bone contact between the segments, as well as condylar placement, have proved to be imprecise or even unfeasible in cases of initially distorted or absent hemimandible due to trauma or pathology; although, there has been some evidence suggesting preplating may allow for appropriate condylar placement (10, 11). Therefore, the concept of VSP was introduced into the preoperative and perioperative workflow of mandibular reconstruction. By using digital surgical simulation and 3D stereolithographic models of the reconstructed mandible, VSP assists in preoperative planning. The concept of computer-generated imaging emerged in the 1990s (12, 13). As the technical possibilities, in particular 3D printing and CAD/CAM devices, have significantly developed over the last years, novel opportunities for intraoperative assistance tools have been proposed. For instance, the utilization of 3D-printed or CAD/CAM-constructed cutting guides for the mandible and fibula, along with patient-specific osteosynthesis plates (PSP), facilitate the shaping process and osteosynthesis of the fibula. Additionally, the inclusion of positioning templates and prepositioned screw holes in the fibula enhances the precision and efficiency of graft positioning.

VSP in combination with personalized CAD/CAM-produced devices was initially developed for saving time and assisting surgeons in mimicking the native mandibular morphology, Benefits of VSP in the setting of FFF reconstruction include: (i) decreased intraoperative time associated with reduced ischemic time of the FFF, (ii) improved accuracy of the reconstruction due to cutting guides and precalculated contact surfaces, (iii) enhanced cosmetic outcomes, (iv) preoperative simulation of postoperative results, reconstruction limitations, and possible complications, (v) presurgical visualization of patient-specific anatomical variations, (vi) clear margins at tumor resection (in cases of tumor resection before FFF reconstruction) (14–18). Among these, increased reconstruction accuracy and decreased intraoperative time proved to be the most significant benefits for enhancing patient outcomes (17, 19–22).

Most studies evaluated accuracy by comparing measurements of preoperative VSP and measurements of postoperative images, assessing the intercondylar distance and gonion angle. A meta-analysis from 2020 including six studies reported an increased accuracy of VSP compared to free-hand surgery. Moreover, the authors found a decreased total operation time of 291.8 min in VSP (vs. 457.6 min in free-hand) and ischemia time of 73.8 min in VSP (vs. 109.9 min in free-hand) (23). Another systemic review of 2019 including 13 studies, reported improved accuracy of VSP when compared to conventional FFF reconstruction techniques. Of note, two studies found no differences in preoperative and postoperative symmetry or functional outcomes (24, 25). Barr et al. described that the total operative time was reduced by 44.64 min in the VSP cohort (6). As operation time constitutes a crucial factor for patient safety and surgical outcome following FFF surgery, perioperative time saving is commonly considered an important surrogate parameter for enhanced patient outcomes (26, 27). Further, a more efficient flap shaping procedure may lead to increased free flap survival rates and overall reduced ischemic time (28, 29). Of note, overall operating time included surgical reconstruction time, as well as the amount of virtual planning time. Thus, saving operation time also strongly depends on the practical experience and skills of the surgeon performing the reconstruction (30). In cases of tumor resection before FFF reconstruction, VSP can help the surgeon understand the spatial relationship of the tumor to adjacent tissue and preserve valuable anatomic structures (e.g., branches of the facial and/or the trigeminal nerve) (14). Further, more individualized reconstructions with more frequent and complex osteotomies can be performed using CAD/CAM-supported VSP (31). Common disadvantages in VSP involve (i) additional costs of VSP equipment, (ii) delay of high-priority surgery due to extended preoperative planning time (15, 16, 32). The extensive costs of VSP in addition to the surgery itself result from expensive VSP software and CAD/CAM or 3D printing devices, outsourcing of working steps to an external digital laboratory, and engagement of specialized personnel (32, 33). To this end, in-house CAD/CAM workflow is considered an alternative to current outsourcing practices (34–36). Here, obtaining adequate funding and the current European Union Medical Device Regulation (EU MDR) as some of the biggest challenges in setting up an in-house 3D-printing core (35). However, once established, an in-house 3D-printing program may reduce costs with no differences in the accuracy of the reconstruction results (37).

### 2.2. The role of artificial intelligence in virtual surgical planning

To date, research efforts are underway to integrate AI into VSP for pattern detection and mean models. However, AI-designed mandibular grafts still play a minor role in everyday clinical work as the final design must be adjusted and refined by surgeons and engineers (38, 39). Although AI is still undergoing a constant process of improvement with increasing data processing capacities for machine learning (ML), AI is already used in preoperative segmentation of the skull CT. While manual segmentation is time-consuming and depends on the surgeon’s subjective setting of Hounsfield unit (HU) thresholds, AI allows for more objective and standardized reconstruction planning (40). Moreover, mandibular deformities caused by neoplasms or congenital anomalies can complicate the understanding and imaging of the patient’s mandibular morphology. In these cases, AI allows for capturing the key features and abnormalities of the mandible and generates authentic images of a typical mandible using generative adversarial networks (GANs) (41). However, the surgeon’s anatomical expertise remains a valuable skill to consider various anatomic key structures, such as the inferior alveolar nerve canal, teeth, condylar head, or glenoid fossa, during VSP. Further, metal artifacts in imaging, due to dental crowns, implants, or orthodontic appliances, can significantly increase the workload of image segmentation, therefore impacting surgical planning, and thus surgical outcomes (42). In this context, AI relies on refinements referring to dependable anatomical skills and image evaluation. Here, ML and deep learning (DL) allow AI to extract information from big data sets to autonomically develop AI’s capacities and enhance the use of AI in VSP (43). Clinical decision-making support (DCS) based on AI recommendations is an upcoming tool in radiology aiming to improve patient safety and value-based imaging (44). In this context, AI could play an important role in the detection and interpretation of incidental findings in radiologic imaging. AI, coupled with advancements in imaging, such as diffusion weighted MRI and radiomics, may lead to significant improvements in patient care, especially for tumor segmentation and perineural invasion, advancing the clinical impact on R0 resections. Further, DL-powered approaches for segmentation of imaging, particularly CT and MRI, were applied in other surgical and non-surgical specialties such as cardiology, neurosurgery, and oncology (45–47). In the field of maxillofacial surgery, current research efforts focus on training AI models in segmentation of the mandibular and maxillary bone, the mandibular canal, the mandibular condyle head, the maxillary sinus, native and treated teeth, as well as implants (48–50). To support accurate direct segmentation of high-resolution skull images regardless of limited graphics processing unit (GPU) memory, Verhelst et al. suggest a two-staged approach that combines one U-Net on a full-size low-resolution image with another U-Net segmenting high-resolution region of interests (51). While DL-supported mandibular segmentation is continuously advancing, technical enhancements in 3D imaging and anatomical accuracy are still required.

## 3. Intraoperative innovations – patient-specific osteosynthesis implants

### 3.1. Patient-specific topology-optimized osteosynthesis plates (TOPOS-implants)

Reconstruction plates are typically used in FFF reconstruction when increased stability is required due to a High load-bearing concept. In contrast to miniplates, adjusting reconstruction plates to the contour of the mandible seems to be more difficult due to higher volume and rigidity (11). Moreover, postoperative plate exposure persists as a common clinical challenge, which necessitates reoperation including plate removal and secondary reconstruction (5, 25, 52–54). Vice versa, miniplates are easily adjustable by the surgeon but are only indicated for load-sharing concepts due to their reduced material thickness (55–57). To improve clinical outcomes following FFF reconstruction and facilitate restoring the mandibular contour, VSP combined with patient-specific osteosynthesis plates have been introduced as a novel therapy concept. Along the preoperative planning process virtual osteosynthesis plates can be fitted and adapted to the surface of the bone segments. 3D-printing techniques allow for on-time fabrication of patient-specific osteosynthesis implants. Thus, this approach results in reduced surgery time and costs, high accuracy levels, and reduction of postoperative complications (58–60). Although the utilization of patient-specific osteosynthesis implants may be expensive, they will reduce operating room time and postoperative complications, and thus, reduce overall healthcare costs for patients and the healthcare system. Current strategies to further improve patient-specific implant (PSI) application in FFF reconstruction describe a novel technique which uses a powerful mathematical tool, called topology optimization (61–63). The generation of an ideal structural design based on preset loading capacities, allows for decreased volume, optimized mechanics, and modular exchangeability of such TOPOS-implants. Thus, TOPOS-implants unify the advantages of reconstruction plates and miniplates by using the material distribution method for topology optimization. The distributed loading of the fixation screws leads to stress shielding and prevents overloading of the screws. A reduced contact area between TOPOS-implant and bone improves the healing capacities due to the reduced periost irritation. Further, the geometric form of the implants also functions as a positioning guide for the bony segments (62). To this end, TOPOS implants represent a high-yield intraoperative approach, exploiting modern technologies and bioengineering concepts with the goal of time and cost efficiency in FFF surgery (64–66). However, topology optimization for designing osteosynthesis plates in maxillofacial surgery have only been described in low-number case series or case reports (67–69). Together with the fact that current research work is limited to biomechanical and cadaveric study designs, further studies are warranted before implementing this approach into the clinical workflow.

### 3.2. How to bypass the need for double-barrel technique? – patient-specific plate

One of the main limitations of FFF reconstruction is the vertical height of the fibula. The fibula, harvested as a single barrel bone, does not exhibit a sufficient diameter to restore the height of the native mandible. This implicates lowered positions of postoperative-inserted implants, and challenges in occlusion, prosthetics, and implantation, while maintaining a better esthetic outcome; on the other hand, higher positions postoperative-inserted implants allow for adequate position of implants and maintenance, but is associated with poorer esthetic outcomes (70, 71). Delayed onlay bone graft, iliac bone reconstruction, fibula distraction, and double-barrel fibula flap graft have been under investigation to address the height issue. Additionally, patient-specific implants (PSI) can be used to reconstruct the original height of the mandible for more extensive defects, such as lower mandibular borders (70). Given the high postoperative predictability, reliable implant outcomes, and conceptualization as a one-stage procedure, double barrel reconstruction is considered the surgical procedure of choice for vertical augmentation in FFF reconstruction, especially for anterior segment reconstruction (18, 72, 73). The morbidity in these procedures is related to the amount of bone left proximally and distally, therefore, it is not advisable to reconstruct defects longer than 10 cm using the double barrel approach (which would require a graft length of about 24 cm), since it may increase the risk for high donor site morbidity (73–76). To overcome the limitation of vertical height in FFF and bypass the downsides of the double barrel approach, a novel technique using 3D-printed PSI has been introduced recently. By modifying the design of a titanium PSI, the plate was intended to support the FFF at an alveolar bone level above the typical inferior mandibular border. A considerable advantage of this technique was the occlusion-derived design of PSI and FFF (i.e., preoperative simulation of an ideal occlusion in terms of VSP). In cases with unilateral edentulous areas, the teeth on the healthy side were mirrored on the defective side to simulate physiological occlusion. If both arches were edentulous, a radiographic guide, including radiopaque teeth, could be used during CT examination to visualize teeth in their maxillomandibular relationship. For patients undergoing these procedures, dentists and maxillofacial surgeons assess occlusion height and determine optimal teeth position, and the FFF is positioned to optimize implant position and maintenance. The PSI plate was constructed with a containing deck to support the bony FFF in a higher position and equally distribute occlusal loading onto the bone screws and the PSI. This can also be used for esthetic purposes, while using miniplates for the fibula. In this study, the PSI-based approach could completely restore the mandibular contour of the inferior border and allowed the surgeon to position bony segments on the alveolar level. It is also possible to pre-plan and include simultaneous implants and immediate placement of dentures. Achieving a more esthetic and feasible contour of the mandible along with better prognosis for prosthetic rehabilitation, this novel technique represented a promising next step in PSI reconstruction. Of note, there were no statistical differences in mechanical properties between 3D-printed titanium plates and conventional surgical plates used in mandibular reconstruction (77). However, disadvantages of this approach were the cost itself, time to conduct surgery, paucity of data about ideal design of the implant, and the risk of exposure, especially after radiotherapy in an oncologic setting (70, 71). Given these limitations, further studies are needed to determine the clinical impact of this concept.

## 4. Back to the future? – augmented reality and bioprinting strategies

### 4.1. Virtual reality and augmented reality

VR is defined as the digitization of objects and environments. To interact with the virtual surgical environment handheld controllers and devices with haptic feedback are used. Currently, VR is applied in preoperative anatomical assessment, VSP, and intraoperative navigation. AR allows projection of 3D objects on the user’s eyesight to create a superimposition of digital content to the real field of view. Thus, additive information about anatomic visualization, preoperative planning, and measurements in the form of holograms can be projected into the surgeon’s operation field (78). This technology was recently adapted by Microsoft® with the “hololens” concept, which allows interactions with the holograms due to cutting-edge hand-motion-recognizing technology (79). Contrary to image-guided navigation and VR, AR renders the need to look outside of the operation field obsolete (80). Consequently, surgical efficiency, assurance of sterility, and immediate data access are supported when using AR. Notably, the intraoperative use of AR devices can also facilitate detailed surgery documentation and assist in legal issues. So far, AR-enhanced surgeries have reached robust levels of accuracy, while showing high rates of preserving critical anatomical structures (81, 82). Although AR has mainly been used in neurosurgery, orthognathic surgery, and facial deformity repairs, previous studies point toward promising results in mandibular reconstruction (especially in complex mandibular reconstructions cases) too (83–89).

Yet, the following limitations delay AR implementation into the FFF reconstruction workflow: The vergence-accommodation conflict is caused by different points of focus and vergence. This phenomenon is due to AR displays being featured by a fixed focal distance, while the ocular point of focusing and verging remain the same (90, 91). Further, errors during the registration and tracking processes of the AR device may lead to misalignments of the virtual objects with the real-life surgery site (92, 93).

### 4.2. Recent advancements in 3D printing

The technology of 3D printing enables the fast-track translation of CAD data into physical 3D models (94). In contrast to CAD/CAM, 3D printing defines various methods of building-up objects in a layer-by-layer fashion (i.e., additive manufacturing). Several methods of 3D printing are currently available, including (i) vat photopolymerization, (ii) powder bed fusion, (iii) material jetting, (iv) material extrusion (v) directed energy deposition, (vii) binder jetting, and (viii) sheet lamination (95, 96). Powder bed fusion, more specifically selective laser melting/sintering (SLM/SLS), is mainly used for constructing titanium PSI in maxillofacial surgery (65, 97, 98). Typically, a stereolithographic resin model/mold is 3D-printed to construct a rapid and reliable prototype. Upon this prototype, the definitive titanium osteosynthesis plate can be flexed, pressed, or molded preoperatively, and most notably, controlled against the stereolithographic model (98). For assisting in surgical planning and checking the fitting accuracy of the PSI preoperatively a polyamide 3D anatomic model of the remaining mandible can be fabricated by means of 3D printing (i.e., SLS) (99). Recent studies and case reports reported high accuracy levels and patient satisfaction following 3D-printed PSI, rendering 3D printing a valuable tool in up-to-date FFF reconstruction (100–102). Furthermore, current locking systems in 3-D printing are milled or printed, which has been shown to be highly accurate and may minimize the need for manual adjustments during the surgery. In addition, in maxillary reconstruction, 3-D printing of Titanium subperiosteal implants has shown improved long term outcomes for patients (103). There is ongoing debate about what material is the most effective bone anchored implant, however, current evidence suggests that both commercially pure titanium (cp-Ti) and titanium alloy (Ti6Al4V) are effective, and there is no clear difference between the two materials (104). 3-D printing has also moved toward utilization of poly ether-ether-ketone (PEEK) and cellular calcium hydroxyapatite (CHAp), which have extraordinary strength and function similar to biological materials, making them an exciting area of growth within this field (105). Advancements have also been shown with resorbable material, such as Magnesium, especially for procedures related to bone tissue repair (106). Finally, research is advancing in harnessing good manufacturing practice level autologous adipose stem cells for maxillary reconstruction (107).

### 4.3. Recent advancements in bioprinting

Bioprinting represents a novel technology, which emerged from the available 3D printing. The concept of integrating living cellular biomaterials into a controlled layer-by-layer deposition allows for the creation of complex, heterogenous tissues with maintained cellular viability (108). Similar to conventional 3D printing strategies, bioprinting is based on data derived from 3D imaging, such as computerized tomography (CT) and magnetic resonance imaging (MRI), translated into CAD files. While there are various bioprinting methods, including inkjet 3D bioprinting, micro-extrusion 3D bioprinting, laser-assisted 3D bioprinting, and stereo-lithography, all approaches rely on a combination of printing biomaterials (i.e., a composition of cells, gels, and growth factors, referred to as bioink), scaffold, and other additive factors (109). Playing a key role in the development of 3D bioprinting, optimizing the composition and texture of bioink represents the pivotal step to bridge the gap from bench to bedside. To organize the cells contained in the bioink biocompatible scaffolds are commonly used as structural support, where cells can adhere, proliferate, and differentiate. The scaffold further includes a plethora of bioactive agents (e.g., interleukins), which stimulate cell growth and proliferation. Cell viability and vascularization are supported by the porous structure of the scaffold (110). While current research is still perplexed by the formation of organic tissue embryonic development, this insight into the embryonic maturation process could represent a promising alternative to using scaffolds for cell organization (111).

**Figure 1:**
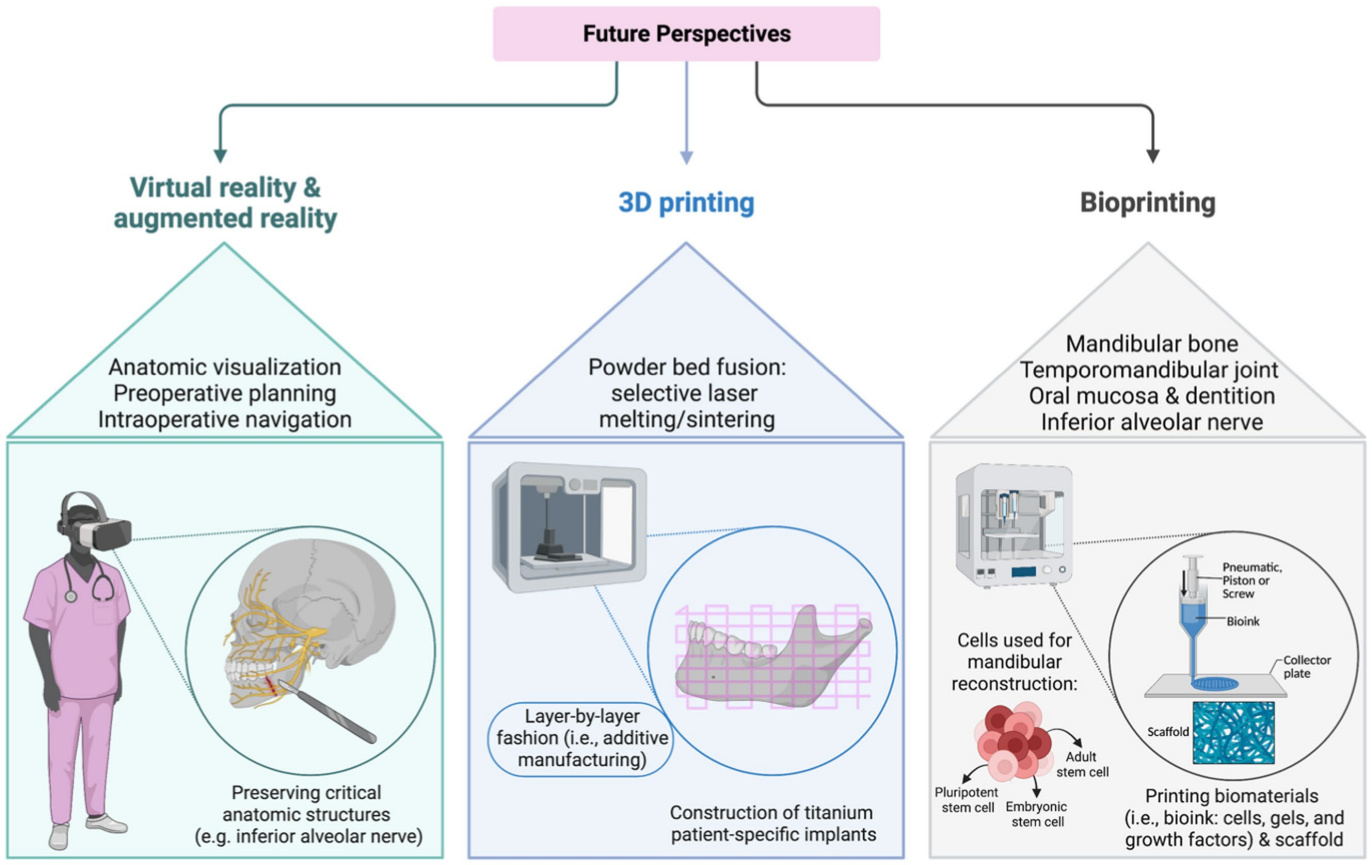
Future perspectives of virtual /augmented reality, three-dimensional (3D), and bioprinting.

Various tissue components have to come from elsewhere in composite FFF transplants: (i) mandibular bone, (ii) temporomandibular joint, (iii) oral mucosa (iv) dentition, and (v) the inferior alveolar nerve. The reconstructed mandibula should resist life-long mastication stress, permanent contractile tension from adjacent facial soft tissue, and the microbiome of the oral cavity. Therefore, bioprinted tissue for mandibular reconstruction must be designed in compliance with mechanical strength, infection resistance, and facial esthetics (112–114). The main components of the mandibular bone are inorganic elements (i.e., hydroxyapatite (HA)), ensuring rigidity, and organic, resilience-ensuring elements (i.e., collagen) (115). Analogically, various scaffold materials have been studied for their inorganic-element-like structure, such as bioceramics of calcium phosphate components (e.g., α-tricalcium phosphate, β-TCP, and HA), synthetic biopolymers (e.g., polycaprolactone, polylactic acid, polylactic glycolic acid, polyethylene glycol), and natural polymers of chitosan, (116–119). Three types of stem cells can be used for mandibular reconstruction: adult stem cells (ASC), embryonic stem cells, and induced pluripotential stem cells (iPSC). Interestingly, mesenchymal stem cells (MSC) obtained from craniofacial bones display a dissenting genetic and histological profile compared to MSC stemming from long bones (114). Further, craniofacial MSC show impaired properties of proliferation and regeneration vs. increased levels of compact bone and alkaline phosphatase (120–123). Currently, there is a mounting body of research investigating further sources of MSC, such as dental pulp, periodontal ligaments, developing teeth, and gingival tissues. For instance, a clinical phase I trial reported promising results in maxillary alveolar cleft reconstruction using deciduous pulp MSC (124). Additional ongoing clinical trials investigate the regenerative potential of gingival fibroblast MSC and dental MSC for tissue engineering (125, 126). Bioactive molecules, such as VEGF, BMP, FGF, and IGF-1 stimulate the angiogenesis and the osteogenic differentiation when incorporated into the bioink (127). Of note, the reconstruction process of a mandibular defect with bioprinting procedures is highly individualizable as the bioprinted graft can be customized to anatomic structures and include a wide composition of biomaterials (128). After uploading 3D image modalities, such as CT, MRT, or PET-CT, onto an on-demand platform, VSP is performed on a multidisciplinary background. At this stage, feasible compositions and configurations of scaffold, cells, and bioactive molecules must be determined. For optimized intraoperative controlled positioning a surgical guide should be designed simultaneously to bioprinting the mandibula. After confirming the final draft, the neo-mandible can be printed in multiple cartridge manners (i.e., integrated tissue and organ printing (ITOP)), transported to the operating room, and finally inserted into the mandibular defect (114). Overall, bioprinted grafts can lower donor site morbidity, reduce operation time, and make graft shaping less sophisticated. Recently, numerous studies have investigated how to optimize both the bioprinting technology and bioink formulas to enhance tissue properties, such as stability and robustness (129–131). However, there is a scarcity of clinical experience on safety and outcomes of bioprinted tissues in maxillofacial reconstruction. Besides a limited number of case reports discussing this technology for the urethra, trachea, and blood vasculature, bioprinted tissue has not been frequently transplanted into human individuals (132). Moreover, there are no clinical trials listed at ClinicalTrails.gov, except for one study investigating the safety and efficacy of traumatic bone defects treated with a 3D tissue-engineered bone equivalent (3D-TEBE) (133). Although the technology of bioprinting still needs to bridge the translational gap for routine clinical use, 3D printed composite tissue is likely to become a versatile tool for maxillofacial, plastic, and head and neck reconstructive surgeons (134). Future perspectives of augmented/virtual reality, as well as 3D- and bioprinting are illustrated in Figure 1.

## 5. Progress in the postoperative period – the advent of modern free flap monitoring

### 5.1. Man vs. machine – novel techniques of flap monitoring

Postoperative complications after FFF reconstruction may occur in about 28–36% of cases. Donor site morbidity is a relatively rare major complication compared to other flap harvest sites, making FFF the gold standard currently, however, there is still risk of other minor complications such as infection, necrosis, fistula, and flap loss (135, 136). Risk factors for postoperative complications include prolonged ischemic times, comorbidities, alcohol withdrawal, smoking, postoperative radiotherapy, and (pre)prosthetic surgery (132, 133). While vascular adverse events typically manifests within 72 h after surgery (i.e., the timeframe during which the anastomosed vessels commonly reepithelialize), inpatient monitoring represents the most powerful tool in flap failure prevention (134). In many cases, a bone reconstruction may be preferred over a 3-D printed load-bearing plate to decrease the risk of plate exposure and for the ability to insert implants followed with dental restoration and regaining masticatory function. In maxillofacial procedures specifically, VSP allows surgeons to perform more accurate reconstructions, allowing dental rehabilitation, and even immediate loading. In case of a skin paddle, the gold standard of flap monitoring comprises clinical examination (i.e., assessing parameters of skin color, temperature, tissue turgor, and capillary refill) and handheld acoustic doppler sonography (ADS) (137, 138). Yet, clinical examination is largely subjective. Therefore, experience and expertise in interpreting the variety of warning symptoms for flap failure are crucial. Further, the specific light source used to inspect the FFF and dark skin tone represent additional pitfalls when evaluating FFF failure (137, 139, 140). Monitoring of buried FFFs is even more challenging as there is no direct access postoperatively to the bony segment and the use of ADS is commonly limited to superficial vessels (141). To overcome such obstacles novel strategies in postoperative free flap monitoring were developed, namely (i) color duplex ultrasonography (CDS), (ii) flow coupler (FC), (iii) implantable doppler (ID), (iv) laser doppler flowmetry (LDF), (v) near-infrared spectroscopy (NIRS), and (vi) hyperspectral imaging (HSI). However, none of these techniques has been established for postoperative FFF management (137). However, several studies underlined the clinical value of CDS as a reliable monitoring instrument, which allows for safe and non-invasive postoperative assessment of buried flaps and vascularized free bone flaps (142, 143). The FC and ID technique are inserted intraoperatively to ensure continuous postoperative monitoring of venous outflow. While ID devices include an external Doppler console and an ultrasonic probe attached to a silicone sheet, which is wrapped around the venous pedicle, FC is not linked to any additional silicone sheet carrying the risk of dislodging. While both FC and ID represent promising tools for monitoring deeply buried flaps allowing for prompt intervention, the high false positive rates of up to 17% slows down their clinical implementation (141, 144, 145). In contrast, LDF, NIRS, and HSI represent non-invasive monitoring alternatives with poor cost-efficiency (146, 147). Currently, there are further promising monitoring techniques underway, which remain to be extensively employed in a clinical setting. For example, the Beckman Laser Institute developed the modulated imaging (MI) tool, a device for detecting changes in a flap appearance due to arterial or venous occlusion, fat necrosis, or flap atrophy. One main advantage of this technique is the early detection of venous occlusion, even before manifestation of other clinical symptoms (148). Further, there are two ongoing studies, evaluating the monitoring of bony free flaps with means of metabolite detection in the interstitial liquid (i.e., microdialysis) (149, 150). Decreased glucose and pyruvate levels vs. increased lactate levels following anaerobic metabolism indicate an impairment of tissue perfusion (e.g., due to ischemia). By placing a microdialysis catheter directly in the bone tissue, the glucose-to-lactate ratio can be determined in bony free flap (151). Since recent studies have proposed optimal positioning sites of the catheter in the surrounding soft tissue, this technique provides more accurate reflection of bone vascularization and represents a promising tool for FFF observation (152, 153). However, further studies are required to identify concrete cut-off values of metabolites in bone tissue (154). Postoperative outcomes, ranging from esthetic outcome to recovery time, have significantly improved as a result of the accuracy of VSP and improvements in surgical planning overall (6, 11, 17, 155, 156).

### 5.2. Artificial intelligence-supported monitoring

**Figure 2:**
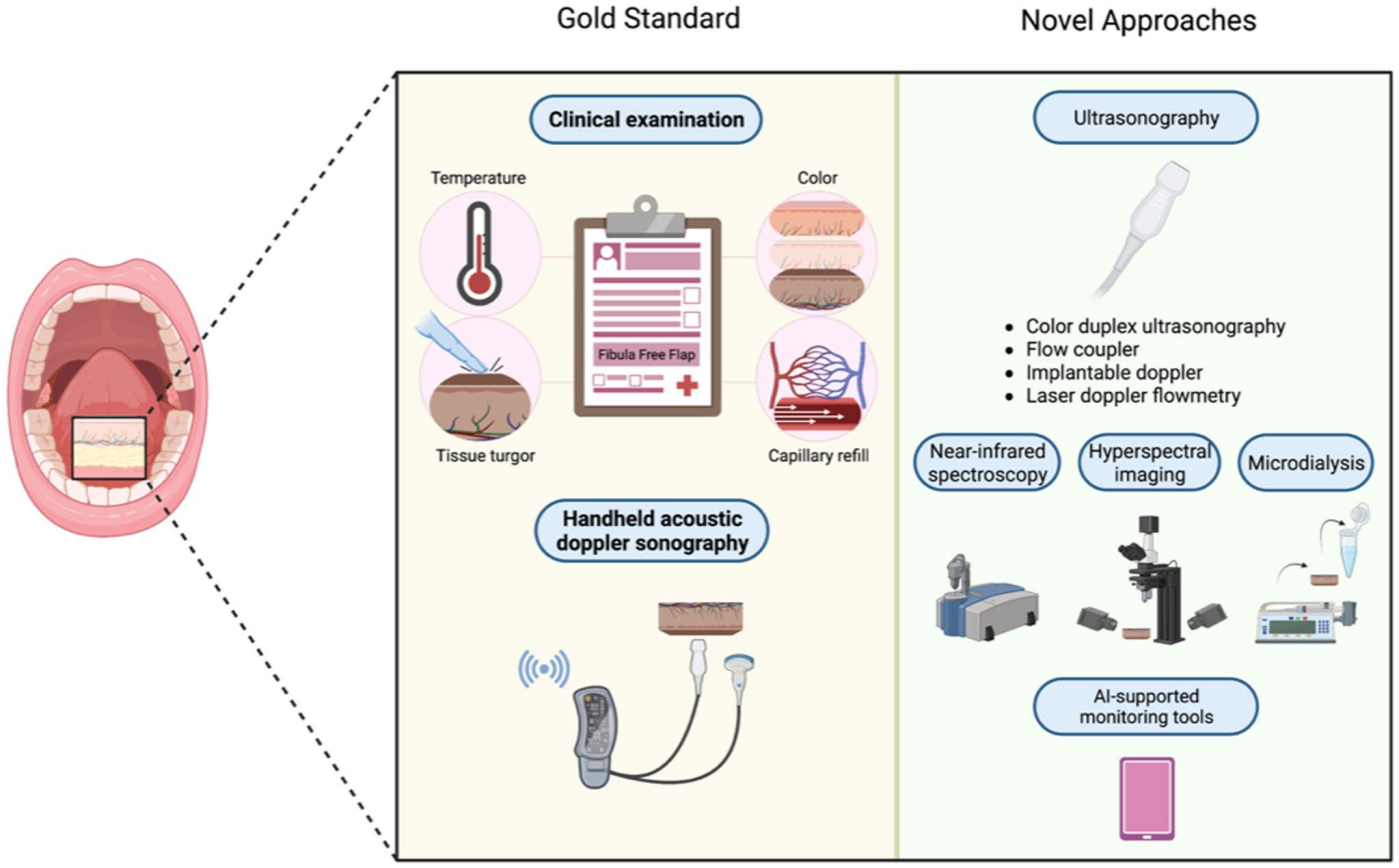
Novel approaches in postoperative monitoring vs. the current gold standard.

While the aforementioned techniques are operator-dependent and subjective, AI is a promising aid in standardizing flap observation and interpretation of continuous flap monitoring (157). Recent literature describes increasing effort in supervising ML tools for postoperative free flap monitoring. Reporting accuracy rates of up to 98.4% AI-supported monitoring tools proved to be time-effective and overcome shortage of specialty-trained clinic staff (158). Moreover, using AI in flap monitoring allows for outsourcing flap assessment from an inpatient setting to home examination. Thus, flap monitoring could be expanded beyond the inpatient period and, vice versa, inpatient time could be shortened, leading to a saving of clinical staff and costs. Notably, the first smartphone application for such microsurgery monitoring was developed back in 2014. Here, varying degrees of pressure were applied around the index finger to produce partial and complete occlusion, which were analyzed by AI based on photographs (159). Another recently developed smartphone-based free flap monitoring tool was described by Provenzano et al., who use ML to develop and validate the assessment model (160, 161). For this purpose, patient data covering various scales of the Fitzpatrick skin type spectrum were utilized to simulate arterial and venous occlusion using a blood pressure tourniquet. Subsequently, a simplistic pattern recognition algorithm was developed to predict arterial or venous occlusion (161). Despite the accuracy and simplicity of utilizing smartphone applications, the EU MDR considers any smartphone application for such purposes as a medical device, making it difficult to get access to such applications. Although AI-supported monitoring tools represent a convenient, economical, and accurate strategy, larger-scale randomized controlled trials are warranted to investigate the clinical implementation of ML devices (162–164). Figure 2 compares novel postoperative monitoring tools to conventional monitoring techniques (Table 1).

**Table 1:** Strengths and limitations of different pre- and intraoperative innovations for fibula free flap surgery.

Technique/Method	Description	Advantages	Limitations
Virtual Surgical Planning (VSP)	Involves using imaging technology (like CT scans) and 3D modeling software to plan surgeries in advance Finds application in complex surgeries where precision is crucial	Decreased intraoperative time Improved accuracy of reconstruction Visualization of predictable results, reconstruction limitations, and possible complications Clear margins at tumor resection	Additional costs Risk of losing familiarity with conventional techniques Delay of surgery due to extended preoperative planning time
Artificial Intelligence (AI) in VSP	Used to create models and help surgeons make decisions based on these models	Timesaving with objective and standardized reconstruction planning Reasonable image generation in case of initial mandibular deformity Enhancement of AI use in VSP with machine learning and deep learning	AI-designed grafts need adjustments and refinement by surgeons and engineers Relies on refinements referring to dependable anatomical skills and image evaluation
Patient-Specific, Topology-Optimized Osteosynthesis Plates (TOPOS-implants)	Represent a type of patient-specific implant that use principles of topology optimization Strive to create an ideal load distribution within the implant	Decreased volume, optimized mechanics, and modular exchangeability Prevents overloading of the screws Reduced contact area between TOPOS-implant and bone improves healing capacities	Current studies are limited to biomechanical and cadaveric investigations
Patient-Specific Plate (PPP)	Depicts a personalized implant designed based on patient’s anatomical data obtained from CT or MRI scans Commonly used for the reconstruction of facial bones after tumor resection or trauma	Overcomes limitations of vertical height in FFF and avoids double barrel procedure Allows for ideal occlusion in terms of VSP Leads to better prognoses for prosthetic rehabilitation	Relatively large amount of titanium needed Risk of plate exposure after radiotherapy Limited availability of studies for treatment of malignant tumors

## 6. Conclusion

FFF is based on an intriguing concept that combines anatomical and clinical strengths resulting in reliable patient outcomes. While FFF has emerged as a high-volume working horse in facial reconstructive surgery, recent advancements in perioperative technologies have paved the way toward innovative and novel concepts for advancing FFF. This review is the to date largest effort to condense the current knowledge of clinical and translational challenges in FFF surgery research and discuss potential solution possibilities. Overall, the integration of cutting-edge technologies into the perioperative workflow of FFF surgery demonstrates promising preliminary results, while carrying untapped potential at the same time. This line of research may guide future research efforts and catalyze the implementation of modern technologies into FFF surgery.

## Author contributions

HB, MA, and LK: conceptualization and writing – original draft. A-FS, MA, and LK: methodology and supervision. HB, CH, SK, and MK-N: investigation. CH, SK, MK-N, A-FS, and BM: writing – review and editing. All authors contributed to the article and approved the submitted version.

## References

[1] Taqi M Raju S. Fibula free flaps. Treasure Island (FL): StatPearls Publishing (2023). 33232007

[2] Kovalenko A Slabkovskaya A Drobysheva N Persin L Drobyshev A Maddalone M. The association between the psychological status and the severity of facial deformity in orthognathic patients. Angle Orthod. (2012) 82:396–402. doi: 10.2319/060211-363.1, PMID: 22007634 PMC8865818

[3] Pruzinsky T. Social and psychological effects of major craniofacial deformity. Cleft Palate Craniofac J. (1992) 29:578–84. doi: 10.1597/1545-1569_1992_029_0578_sapeom_2.3.co_2, PMID: 1450200

[4] Pellini R Mercante G Spriano G. Step-by-step mandibular reconstruction with free fibula flap modelling. Acta Otorhinolaryngol Ital. (2012) 32:405–9. PMID: 23349561 PMC3552535

[5] Ritschl LM Mücke T Hart D Unterhuber T Kehl V Wolff KD. Retrospective analysis of complications in 190 mandibular resections and simultaneous reconstructions with free fibula flap, iliac crest flap or reconstruction plate: a comparative single Centre study. Clin Oral Investig. (2021) 25:2905–14. doi: 10.1007/s00784-020-03607-8, PMID: 33025147 PMC8060197

[6] Barr M. Virtual surgical planning for mandibular reconstruction with the fibula free flap: a systematic review and Meta-analysis. Ann Plast Surg. (2020) 84:117–22. doi: 10.1097/SAP.0000000000002006 31633539

[7] Hidalgo DA. Fibula free flap: a new method of mandible reconstruction. Plast Reconstr Surg. (1989) 84:71–9. 2734406

[8] Marchetti C Bianchi A Mazzoni S Cipriani R Campobassi A. Oromandibular reconstruction using a fibula osteocutaneous free flap: four different "preplating" techniques. Plast Reconstr Surg. (2006) 118:643–51. doi: 10.1097/01.prs.0000233211.54505.9a, PMID: 16932172

[9] Moro A Cannas R Boniello R Gasparini G Pelo S. Techniques on modeling the vascularized free fibula flap in mandibular reconstruction. J Craniofac Surg. (2009) 20:1571–3. doi: 10.1097/SCS.0b013e3181b0db5c 19816298

[10] Thankappan K Trivedi NP Subash P Pullara SK Peter S Kuriakose MA. Three-dimensional computed tomography-based contouring of a free fibula bone graft for mandibular reconstruction. J Oral Maxillofac Surg. (2008) 66:2185–92. doi: 10.1016/j.joms.2008.01.035, PMID: 18848124

[11] Roser SM Ramachandra S Blair H Grist W Carlson GW Christensen AM. The accuracy of virtual surgical planning in free fibula mandibular reconstruction: comparison of planned and final results. J Oral Maxillofac Surg. (2010) 68:2824–32. doi: 10.1016/j.joms.2010.06.177, PMID: 20828910

[12] Rose EH Norris MS Rosen JM. Application of high-tech three-dimensional imaging and computer-generated models in complex facial reconstructions with vascularized bone grafts. Plast Reconstr Surg. (1993) 91:252–64. 8304990 10.1097/00006534-199302000-00007

[13] Polley JW Cohen M. Three-dimensional imaging and computer-generated models in complex facial reconstructions. Plast Reconstr Surg. (1993) 92:1204–5. doi: 10.1097/00006534-199311000-00054, PMID: 8234529

[14] Myers PL Nelson JA Rosen EB Allen RJ Disa JJ Matros E. Virtual surgical planning for oncologic mandibular and maxillary reconstruction. Plast Reconstr Surg Glob Open. (2021) 9:e3672. doi: 10.1097/GOX.0000000000003672, PMID: 34548995 PMC8448079

[15] Antúnez-Conde R Salmerón JI Díez-Montiel A Agea M Gascón D Sada Á. Mandibular reconstruction with fibula flap and dental implants through virtual surgical planning and three different techniques: double-barrel flap, implant dynamic navigation and CAD/CAM mesh with iliac crest graft. Frontiers. Oncology. (2021) 11:11. doi: 10.3389/fonc.2021.719712 PMC8525397 34676161

[16] Yu Y Zhang WB Wang Y Liu XJ Guo CB Peng X. A revised approach for mandibular reconstruction with the vascularized iliac crest flap using virtual surgical planning and surgical navigation. J Oral Maxillofac Surg. (2016) 74:1285.e1. doi: 10.1016/j.joms.2016.02.021 27019415

[17] Rodby KA Turin S Jacobs RJ Cruz JF Hassid VJ Kolokythas A. Advances in oncologic head and neck reconstruction: systematic review and future considerations of virtual surgical planning and computer aided design/computer aided modeling. J Plast Reconstr Aesthet Surg. (2014) 67:1171–85. doi: 10.1016/j.bjps.2014.04.038 24880575

[18] Navarro Cuéllar C Ochandiano Caicoya S Navarro Cuéllar I Valladares Pérez S Fariña Sirandoni R Antúnez-Conde R. Vertical ridge augmentation of fibula flap in mandibular reconstruction: a comparison between vertical distraction, double-barrel flap and iliac crest graft. J Clin Med. (2020) 10:101. doi: 10.3390/jcm10010101, PMID: 33396707 PMC7795399

[19] Ayoub N Ghassemi A Rana M Gerressen M Riediger D Hölzle F. Evaluation of computer-assisted mandibular reconstruction with vascularized iliac crest bone graft compared to conventional surgery: a randomized prospective clinical trial. Trials. (2014) 15:114. doi: 10.1186/1745-6215-15-114, PMID: 24716651 PMC3998950

[20] Bao T He J Yu C Zhao W Lin Y Wang H. Utilization of a pre-bent plate-positioning surgical guide system in precise mandibular reconstruction with a free fibula flap. Oral Oncol. (2017) 75:133–9. doi: 10.1016/j.oraloncology.2017.11.011, PMID: 29224810

[21] Ren W Gao L Li S Chen C Li F Wang Q. Virtual planning and 3D printing modeling for mandibular reconstruction with fibula free flap. Med Oral Patol Oral Cir Bucal. (2018) 23:e359–66. doi: 10.4317/medoral.22295, PMID: 29680849 PMC5945234

[22] Yu Y Zhang WB Liu XJ Guo CB Yu GY Peng X. Three-dimensional accuracy of virtual planning and surgical navigation for mandibular reconstruction with free fibula flap. J Oral Maxillofac Surg. (2016) 74:1503.e1–1503.e10. doi: 10.1016/j.joms.2016.02.020 27000408

[23] Pucci R Weyh A Smotherman C Valentini V Bunnell A Fernandes R. Accuracy of virtual planned surgery versus conventional free-hand surgery for reconstruction of the mandible with osteocutaneous free flaps. Int J Oral Maxillofac Surg. (2020) 49:1153–61. doi: 10.1016/j.ijom.2020.02.018 32197824

[24] De Maesschalck T Courvoisier DS Scolozzi P. Computer-assisted versus traditional freehand technique in fibular free flap mandibular reconstruction: a morphological comparative study. Eur Arch Otorhinolaryngol. (2017) 274:517–26. doi: 10.1007/s00405-016-4246-4, PMID: 27501991

[25] Ritschl LM Mücke T Fichter A Güll FD Schmid C Duc JMP. Functional outcome of CAD/CAM-assisted versus conventional microvascular, fibular free flap reconstruction of the mandible: a retrospective study of 30 cases. J Reconstr Microsurg. (2017) 33:281–91. doi: 10.1055/s-0036-1597823, PMID: 28099975

[26] Cheng H Clymer JW Po-Han Chen B Sadeghirad B Ferko NC Cameron CG. Prolonged operative duration is associated with complications: a systematic review and meta-analysis. J Surg Res. (2018) 229:134–44. doi: 10.1016/j.jss.2018.03.022 29936980

[27] Hardy KL Davis KE Constantine RS Chen M Hein R Jewell JL. The impact of operative time on complications after plastic surgery: a multivariate regression analysis of 1753 cases. Aesthet Surg J. (2014) 34:614–22. doi: 10.1177/1090820X14528503, PMID: 24696297

[28] Zhang L Liu Z Li B Yu H Shen SG Wang X. Evaluation of computer-assisted mandibular reconstruction with vascularized fibular flap compared to conventional surgery. Oral Surg Oral Med Oral Pathol Oral Radiol. (2016) 121:139–48. doi: 10.1016/j.oooo.2015.10.005, PMID: 26792754

[29] Chang EI Jenkins MP Patel SA Topham NS. Long-term operative outcomes of preoperative computed tomography–guided virtual surgical planning for osteocutaneous free flap mandible reconstruction. Plast Reconstr Surg. (2016) 137:619–23. doi: 10.1097/01.prs.0000475796.61855.a7, PMID: 26818299

[30] Rustemeyer J Sari-Rieger A Melenberg A Busch A. Comparison of intraoperative time measurements between osseous reconstructions with free fibula flaps applying computer-aided designed/computer-aided manufactured and conventional techniques. Oral Maxillofac Surg. (2015) 19:293–300. doi: 10.1007/s10006-015-0493-6, PMID: 25861911

[31] Shenaq DS Matros E. Virtual planning and navigational technology in reconstructive surgery. J Surg Oncol. (2018) 118:845–52. doi: 10.1002/jso.25255 30293247

[32] Huang MF Alfi D Alfi J Huang AT. The use of patient-specific implants in oral and maxillofacial surgery. Oral and Maxillofacial Surgery Clinics. (2019) 31:593–600. doi: 10.1016/j.coms.2019.07.010 31481289

[33] Kurlander DE. The cost utility of virtual surgical planning and computer-assisted design/computer-assisted manufacturing in mandible reconstruction using the free fibula Osteocutaneous flap. J Reconstr Microsurg. (2022) 39:221–30. doi: 10.1055/s-0042-1755260 35988577

[34] Numajiri T Morita D Yamochi R Nakamura H Tsujiko S Sowa Y. Does an in-house computer-aided design/computer-aided manufacturing approach contribute to accuracy and time shortening in mandibular reconstruction? J Craniofac Surg. (2020) 31:1928–32. doi: 10.1097/SCS.0000000000006699, PMID: 32649531

[35] Daoud GE Pezzutti DL Dolatowski CJ Carrau RL Pancake M Herderick E. Establishing a point-of-care additive manufacturing workflow for clinical use. J Mater Res. (2021) 36:3761–80. doi: 10.1557/s43578-021-00270-x, PMID: 34248272 PMC8259775

[36] Khonsari RH Adam J Benassarou M Bertin H Billotet B Bouaoud J. In-house 3D printing: why, when, and how? Overview of the national French good practice guidelines for in-house 3D-printing in maxillo-facial surgery, stomatology, and oral surgery. J Stomatol Oral Maxillofac Surg. (2021) 122:458–61. doi: 10.1016/j.jormas.2021.08.002, PMID: 34400375

[37] Moe J Foss J Herster R Powell C Helman J Ward BB. An in-house computer-aided design and computer-aided manufacturing workflow for maxillofacial free flap reconstruction is associated with a low cost and high accuracy. J Oral Maxillofac Surg. (2021) 79:227–36. doi: 10.1016/j.joms.2020.07.216, PMID: 32860748

[38] Modabber A Rauen A Ayoub N Möhlhenrich SC Peters F Kniha K. Evaluation of a novel algorithm for automating virtual surgical planning in mandibular reconstruction using fibula flaps. J Craniomaxillofac Surg. (2019) 47:1378–86. doi: 10.1016/j.jcms.2019.06.013, PMID: 31331845

[39] Raith S Wolff S Steiner T Modabber A Weber M Hölzle F. Planning of mandibular reconstructions based on statistical shape models. Int J Comput Assist Radiol Surg. (2017) 12:99–112. doi: 10.1007/s11548-016-1451-y, PMID: 27393280

[40] Yang WF Zhang CY Choi WS Zhu WY Li DTS Chen XS. A novel ‘surgeon-dominated’ approach to the design of 3D-printed patient-specific surgical plates in mandibular reconstruction: a proof-of-concept study. Int J Oral Maxillofac Surg. (2020) 49:13–21. doi: 10.1016/j.ijom.2019.05.005, PMID: 31230767

[41] Liang Y Huan JJ Li JD Jiang CH Fang CY Liu YG. Use of artificial intelligence to recover mandibular morphology after disease. Sci Rep. (2020) 10:16431. doi: 10.1038/s41598-020-73394-5, PMID: 33009429 PMC7532179

[42] Yang W-F Su Y-X. Artificial intelligence-enabled automatic segmentation of skull CT facilitates computer-assisted craniomaxillofacial surgery. Oral Oncol. (2021) 118:105360. doi: 10.1016/j.oraloncology.2021.105360, PMID: 34045151

[43] Feng J Phillips RV Malenica I Bishara A Hubbard AE Celi LA. Clinical artificial intelligence quality improvement: towards continual monitoring and updating of AI algorithms in healthcare. NPJ Digit Med. (2022) 5:66. doi: 10.1038/s41746-022-00611-y 35641814 PMC9156743

[44] Bizzo BC Almeida RR Michalski MH Alkasab TK. Artificial intelligence and clinical decision support for radiologists and referring providers. J Am Coll Radiol. (2019) 16:1351–6. doi: 10.1016/j.jacr.2019.06.010, PMID: 31492414

[45] Chen C Qin C Qiu H Tarroni G Duan J Bai W. Deep learning for cardiac image segmentation: a review. Front Cardiovasc Med. (2020) 7:25. doi: 10.3389/fcvm.2020.00025, PMID: 32195270 PMC7066212

[46] Shen L. Anderson T.. Multimodal brain MRI tumor segmentation via convolutional neural networks. (2017). 18: p. 2014–2015. New York, NY: Elsevier.

[47] Christ PF. Automatic liver and tumor segmentation of CT and MRI volumes using cascaded fully convolutional neural networks. arXiv preprint arXiv. (2017) 1702:05970. doi: 10.48550/arXiv.1702.05970

[48] Järnstedt J Sahlsten J Jaskari J Kaski K Mehtonen H Lin Z. Comparison of deep learning segmentation and multigrader-annotated mandibular canals of multicenter CBCT scans. Sci Rep. (2022) 12:18598. doi: 10.1038/s41598-022-20605-w 36329051 PMC9633839

[49] Cha JY Yoon HI Yeo IS Huh KH Han JS. Panoptic segmentation on panoramic radiographs: deep learning-based segmentation of various structures including maxillary sinus and Mandibular Canal. J Clin Med. (2021) 10:2577. doi: 10.3390/jcm10122577, PMID: 34208024 PMC8230590

[50] Kim YH Shin JY Lee A Park S Han SS Hwang HJ. Automated cortical thickness measurement of the mandibular condyle head on CBCT images using a deep learning method. Sci Rep. (2021) 11:14852. doi: 10.1038/s41598-021-94362-7, PMID: 34290333 PMC8295413

[51] Verhelst P-J Smolders A Beznik T Meewis J Vandemeulebroucke A Shaheen E. Layered deep learning for automatic mandibular segmentation in cone-beam computed tomography. J Dent. (2021) 114:103786. doi: 10.1016/j.jdent.2021.103786, PMID: 34425172

[52] Nicholson RE Schuller DE Forrest LA Mountain RE Ali T Young D. Factors involved in long-and short-term mandibular plate exposure. Arch Otolaryngol Head & Neck Surgery. (1997) 123:217–22. doi: 10.1001/archotol.1997.01900020107016, PMID: 9046293

[53] Maurer P Eckert AW Kriwalsky MS Schubert J. Scope and limitations of methods of mandibular reconstruction: a long-term follow-up. Br J Oral Maxillofac Surg. (2010) 48:100–4. doi: 10.1016/j.bjoms.2009.07.005, PMID: 19647911

[54] Wood CB Shinn JR Amin SN Rohde SL Sinard RJ. Risk of plate removal in free flap reconstruction of the mandible. Oral Oncol. (2018) 83:91–5. doi: 10.1016/j.oraloncology.2018.06.008, PMID: 30098784

[55] Malata CM McLean NR Alvi R McKiernan MV Milner RH Piggot TA. An evaluation of the Würzburg titanium miniplate osteosynthesis system for mandibular fixation. Br J Plast Surg. (1997) 50:26–32. doi: 10.1016/S0007-1226(97)91279-0, PMID: 9038511

[56] Evans GR Clark N Manson PN Leipziger LS. Role of mini- and microplate fixation in fractures of the midface and mandible. Ann Plast Surg. (1995) 34:453–6. doi: 10.1097/00000637-199505000-00001, PMID: 7639480

[57] Robey AB Spann ML McAuliff TM Meza JL Hollins RR Johnson PJ. Comparison of miniplates and reconstruction plates in fibular flap reconstruction of the mandible. Plast Reconstr Surg. (2008) 122:1733–8. doi: 10.1097/PRS.0b013e31818a9ac5, PMID: 19050525

[58] Mascha F Winter K Pietzka S Heufelder M Schramm A Wilde F. Accuracy of computer-assisted mandibular reconstructions using patient-specific implants in combination with CAD/CAM fabricated transfer keys. J Craniomaxillofac Surg. (2017) 45:1884–97. doi: 10.1016/j.jcms.2017.08.028, PMID: 28965991

[59] Dérand P III Rännar L-E Hirsch J-M. Imaging, virtual planning, design, and production of patient-specific implants and clinical validation in craniomaxillofacial surgery. Craniomaxillofac Trauma Reconstr. (2012) 5:137–43. doi: 10.1055/s-0032-1313357, PMID: 23997858 PMC3578652

[60] Probst FA Metzger M Ehrenfeld M Cornelius CP. Computer-assisted designed and manufactured procedures facilitate the lingual application of mandible reconstruction plates. J Oral Maxillofac Surg. (2016) 74:1879–95. doi: 10.1016/j.joms.2016.03.015, PMID: 27087284

[61] Bendsoe MP Sigmund O. Topology optimization: Theory, methods, and applications. New York: Springer Science & Business Media (2003).

[62] Lang JJ Bastian M Foehr P Seebach M Weitz J von Deimling C. Improving mandibular reconstruction by using topology optimization, patient specific design and additive manufacturing?—a biomechanical comparison against miniplates on human specimen. PLoS One. (2021) 16:e0253002. doi: 10.1371/journal.pone.0253002, PMID: 34101755 PMC8186800

[63] Seebach M. Design of bone plates for mandibular reconstruction using topology and shape optimization. in Advances in Structural and Multidisciplinary Optimization: *Proceedings of the 12th World Congress of Structural and Multidisciplinary Optimization (WCSMO12)*. 12. (2018). Springer.

[64] Tarsitano A Battaglia S Crimi S Ciocca L Scotti R Marchetti C. Is a computer-assisted design and computer-assisted manufacturing method for mandibular reconstruction economically viable? J Craniomaxillofac Surg. (2016) 44:795–9. doi: 10.1016/j.jcms.2016.04.003 27193477

[65] Darwich K Ismail MB al-Mozaiek MYAS Alhelwani A. Reconstruction of mandible using a computer-designed 3D-printed patient-specific titanium implant: a case report. Oral Maxillofac Surg. (2021) 25:103–11. doi: 10.1007/s10006-020-00889-w, PMID: 32725572

[66] Ciocca L Mazzoni S Fantini M Persiani F Baldissara P Marchetti C. A CAD/CAM-prototyped anatomical condylar prosthesis connected to a custom-made bone plate to support a fibula free flap. Med Biol Eng Comput. (2012) 50:743–9. doi: 10.1007/s11517-012-0898-4, PMID: 22447348

[67] Seebach M Fritz C Kerschreiter J Zaeh MF. Shape accuracy and surface quality of additively manufactured, optimized, patient-specific bone plates. J Medical Devices. (2021) 15:21–8. doi: 10.1115/1.4049193

[68] Koper DC Leung CAW Smeets LCP Laeven PFJ Tuijthof GJM Kessler PAWH. Topology optimization of a mandibular reconstruction plate and biomechanical validation. J Mech Behav Biomed Mater. (2021) 113:104157. doi: 10.1016/j.jmbbm.2020.104157 33187871

[69] Li C-H Wu C-H Lin C-L. Design of a patient-specific mandible reconstruction implant with dental prosthesis for metal 3D printing using integrated weighted topology optimization and finite element analysis. J Mech Behav Biomed Mater. (2020) 105:103700. doi: 10.1016/j.jmbbm.2020.103700, PMID: 32279847

[70] Tarsitano A Battaglia S Corinaldesi G Marchetti C Pellegrino G Ciocca L. Mandibular reconstruction using a new design for a patient-specific plate to support a fibular free flap and avoid double-barrel technique. Acta Otorhinolaryngol Ital. (2021) 41:230–5. doi: 10.14639/0392-100X-N0549, PMID: 34264916 PMC8283401

[71] Tarsitano A Ceccariglia F Bevini M Breschi L Felice P Marchetti C. Prosthetically guided mandibular reconstruction using a fibula free flap: three-dimensional Bologna plate, an alternative to the double-barrel technique. Int J Oral Maxillofac Surg. (2023) 52:436–41. doi: 10.1016/j.ijom.2022.08.006, PMID: 36038455

[72] Klesper B Lazar F Siessegger M Hidding J Zöller JE. Vertical distraction osteogenesis of fibula transplants for mandibular reconstruction–a preliminary study. J Craniomaxillofac Surg. (2002) 30:280–5. doi: 10.1016/S1010-5182(02)90315-X, PMID: 12377200

[73] Lizio G Corinaldesi G Pieri F Marchetti C. Problems with dental implants that were placed on vertically distracted fibular free flaps after resection: a report of six cases. Br J Oral Maxillofac Surg. (2009) 47:455–60. doi: 10.1016/j.bjoms.2009.06.002, PMID: 19576667

[74] Sinclair C Gleysteen JP Zimmermann TM Wax MK Givi B Schneider D. Assessment of donor site morbidity for free radial forearm osteocutaneous flaps. Microsurgery. (2012) 32:255–60. doi: 10.1002/micr.21950, PMID: 22473601 PMC3951340

[75] Bähr W Stoll P Wächter R. Use of the “double barrel” free vascularized fibula in mandibular reconstruction. J Oral Maxillofac Surg. (1998) 56:38–44. 9437980 10.1016/s0278-2391(98)90914-4

[76] Anne-Gaëlle B Samuel S Julie B Renaud L Pierre B. Dental implant placement after mandibular reconstruction by microvascular free fibula flap: current knowledge and remaining questions. Oral Oncol. (2011) 47:1099–104. doi: 10.1016/j.oraloncology.2011.07.016, PMID: 21873106

[77] Wang CF Yu Y Bai W Han JM Zhang WB Peng X. Mechanical properties of three-dimensionally printed titanium plates used in jaw reconstruction: preliminary study. Int J Oral Maxillofac Surg. (2022) 51:754–61. doi: 10.1016/j.ijom.2021.09.008, PMID: 34629260

[78] Cofano F di Perna G Bozzaro M Longo A Marengo N Zenga F. Augmented reality in medical practice: from spine surgery to remote assistance. Front Surgery. (2021) 8:8. doi: 10.3389/fsurg.2021.657901 PMC8042331 33859995

[79] Palumbo A. Microsoft HoloLens 2 in medical and healthcare context: state of the art and future prospects. Sensors (Basel). (2022) 22:7709. doi: 10.3390/s22207709, PMID: 36298059 PMC9611914

[80] De Paolis LT De Luca V. Augmented visualization with depth perception cues to improve the surgeon's performance in minimally invasive surgery. Med Biol Eng Comput. (2019) 57:995–1013. doi: 10.1007/s11517-018-1929-6, PMID: 30511205

[81] Badiali G Roncari A Bianchi A Taddei F Marchetti C Schileo E. Navigation in orthognathic surgery: 3D accuracy. Facial Plast Surg. (2015) 31:463–73. doi: 10.1055/s-0035-1564716, PMID: 26579862

[82] Cercenelli L Carbone M Condino S Cutolo F Marcelli E Tarsitano A. The wearable VOSTARS system for augmented reality-guided surgery: preclinical phantom evaluation for high-precision maxillofacial tasks. J Clin Med. (2020) 9:3562–69. doi: 10.3390/jcm9113562, PMID: 33167432 PMC7694536

[83] Zhao R Zhu Z Shao L Meng F Lei Z Li X. Augmented reality guided in reconstruction of mandibular defect with fibular flap: a cadaver study. J Stomatol Oral Maxillofac Surg. (2023) 124:101318. doi: 10.1016/j.jormas.2022.10.017, PMID: 36280109

[84] Meola A Cutolo F Carbone M Cagnazzo F Ferrari M Ferrari V. Augmented reality in neurosurgery: a systematic review. Neurosurg Rev. (2017) 40:537–48. doi: 10.1007/s10143-016-0732-9, PMID: 27154018 PMC6155988

[85] Mischkowski RA Zinser MJ Kübler AC Krug B Seifert U Zöller JE. Application of an augmented reality tool for maxillary positioning in orthognathic surgery–a feasibility study. J Craniomaxillofac Surg. (2006) 34:478–83. doi: 10.1016/j.jcms.2006.07.862, PMID: 17157519

[86] Fushima K Kobayashi M. Mixed-reality simulation for orthognathic surgery. Maxillofacial Plas Reconstruct Surgery. (2016) 38:1–12. doi: 10.1186/s40902-016-0059-z PMC4783436 27014664

[87] Qu M Hou Y Xu Y Shen C Zhu M Xie L. Precise positioning of an intraoral distractor using augmented reality in patients with hemifacial microsomia. J Craniomaxillofac Surg. (2015) 43:106–12. doi: 10.1016/j.jcms.2014.10.019, PMID: 25465484

[88] Gao Y Liu K Lin L Wang X Xie L. Use of augmented reality navigation to optimise the surgical management of craniofacial fibrous dysplasia. Br J Oral Maxillofac Surg. (2022) 60:162–7. doi: 10.1016/j.bjoms.2021.03.011, PMID: 34930644

[89] Pietruski P Majak M Świątek-Najwer E Żuk M Popek M Jaworowski J. Supporting fibula free flap harvest with augmented reality: a proof-of-concept study. Laryngoscope. (2020) 130:1173–9. doi: 10.1002/lary.28090, PMID: 31132152

[90] Zhou Y Zhang J Fang F. Vergence-accommodation conflict in optical see-through display: review and prospect. Results in Optics. (2021) 5:100160. doi: 10.1016/j.rio.2021.100160

[91] Erkelens I.M. MacKenzie K.J.. 19–2: Vergence-Accommodation Conflicts in Augmented Reality: Impacts on Perceived Image Quality. in *SID Symposium Digest of Technical Papers*. (2020). Wiley Online Library.

[92] Chen Y Wang Q Chen H Song X Tang H Tian M. An overview of augmented reality technology. J Phys Conf Ser. (2019) 1237:022082. doi: 10.1088/1742-6596/1237/2/022082

[93] Barcali E Iadanza E Manetti L Francia P Nardi C Bocchi L. Augmented reality in surgery: a scoping review. App Sci. (2022) 12:6890. doi: 10.3390/app12146890

[94] Croke L. Use of 3D printing in surgery. AORN J. (2021) 113:P7–9. doi: 10.1002/aorn.13437, PMID: 34048049

[95] Willson K Atala A. Medical 3D printing: tools and techniques, today and tomorrow. Ann Rev Chemical and Biomolecular Engineer. (2022) 13:481–99. doi: 10.1146/annurev-chembioeng-092220-015404, PMID: 35385675

[96] Kalaskar DM. 3D printing in medicine. United Kingdom: Woodhead Publishing (2022).

[97] Hatamleh MM Bhamrah G Ryba F Mack G Huppa C. Simultaneous computer-aided design/computer-aided manufacture bimaxillary orthognathic surgery and mandibular reconstruction using selective-laser sintered titanium implant. J Craniofac Surg. (2016) 27:1810–4. doi: 10.1097/SCS.0000000000003039, PMID: 27548831

[98] Goodson AMC Kittur MA Evans PL Williams EM. Patient-specific, printed titanium implants for reconstruction of mandibular continuity defects: a systematic review of the evidence. J Craniomaxillofac Surg. (2019) 47:968–76. doi: 10.1016/j.jcms.2019.02.010, PMID: 30885527

[99] Fernandes N van den Heever J Hoek K Booysen G. Customized reconstruction of an extensive mandibular defect: a clinical report. J Prosthet Dent. (2016) 116:928–31. doi: 10.1016/j.prosdent.2016.04.012 27422227

[100] Bedogni A Bettini G Bedogni G Menapace G Sandi A Michelon F. Safety of boneless reconstruction of the mandible with a CAD/CAM designed titanium device: the replica cohort study. Oral Oncol. (2021) 112:105073. doi: 10.1016/j.oraloncology.2020.105073, PMID: 33160150

[101] Schottey O Huys SEF van Lenthe GH Mommaerts MY Vander Sloten J. Development of a topologically optimized patient-specific mandibular reconstruction implant for a Brown class II defect. Ann 3D Printed Med. (2023) 10:100107. doi: 10.1016/j.stlm.2023.100107

[102] Rana M Chin SJ Muecke T Kesting M Groebe A Riecke B. Increasing the accuracy of mandibular reconstruction with free fibula flaps using functionalized selective laser-melted patient-specific implants: a retrospective multicenter analysis. J Craniomaxillofac Surg. (2017) 45:1212–9. doi: 10.1016/j.jcms.2017.04.003, PMID: 28552201

[103] Mangano C Bianchi A Mangano FG Dana J Colombo M Solop I. Custom-made 3D printed subperiosteal titanium implants for the prosthetic restoration of the atrophic posterior mandible of elderly patients: a case series. 3D Print Med. (2020) 6:1. doi: 10.1186/s41205-019-0055-x, PMID: 31915946 PMC6950914

[104] Shah F Trobos M Thomsen P Palmquist A. Commercially pure titanium (cp-ti) versus titanium alloy (Ti6Al4V) materials as bone anchored implants - is one truly better than the other? Mater Sci Eng C. (2016) 62:960–6. doi: 10.1016/j.msec.2016.01.032, PMID: 26952502

[105] Oladapo B Zahedi SA Ismail SO Omigbodun FT. 3D printing of PEEK and its composite to increase biointerfaces as a biomedical material- a review. Colloids Surf B Biointerfaces. (2021) 203:111726. doi: 10.1016/j.colsurfb.2021.111726, PMID: 33865088

[106] Kazakova G Safronova T Golubchikov D Shevtsova O Rau JV. Resorbable Mg2+-containing phosphates for bone tissue repair. Dent Mater. (2021) 14:4857. doi: 10.3390/ma14174857, PMID: 34500951 PMC8432688

[107] Mesimäki K Lindroos B Törnwall J Mauno J Lindqvist C Kontio R. Novel maxillary reconstruction with ectopic bone formation by GMP adipose stem cells. Int J Oral Maxillofac Surg. (2019) 38:201–9. doi: 10.1016/j.ijom.2009.01.001 19168327

[108] Ramadan Q Zourob M. 3D bioprinting at the frontier of regenerative medicine, pharmaceutical, and food industries. Front Medical Technol. (2021) 2:2. doi: 10.3389/fmedt.2020.607648 PMC8757855 35047890

[109] Kim JH Yoo JJ Lee SJ. Three-dimensional cell-based bioprinting for soft tissue regeneration. Tissue Engineer Regen Med. (2016) 13:647–62. doi: 10.1007/s13770-016-0133-8, PMID: 30603446 PMC6170860

[110] Mironov V Visconti RP Kasyanov V Forgacs G Drake CJ Markwald RR. Organ printing: tissue spheroids as building blocks. Biomaterials. (2009) 30:2164–74. doi: 10.1016/j.biomaterials.2008.12.084, PMID: 19176247 PMC3773699

[111] Marga F Neagu A Kosztin I Forgacs G. Developmental biology and tissue engineering. Birth Defects Res C Embryo Today. (2007) 81:320–8. doi: 10.1002/bdrc.20109 18228266

[112] Lin P-Y Lin KC Jeng S-F. Oromandibular reconstruction: the history, operative options and strategies, and our experience. Int Scholarly Res Notices. (2011) 2011:1–10. doi: 10.5402/2011/824251 PMC3246309 22229103

[113] Antony AK Chen WF Kolokythas A Weimer KA Cohen MN. Use of virtual surgery and stereolithography-guided osteotomy for mandibular reconstruction with the free fibula. Plast Reconstr Surg. (2011) 128:1080–4. doi: 10.1097/PRS.0b013e31822b6723, PMID: 22030490

[114] Park HI Lee JH Lee SJ. The comprehensive on-demand 3D bio-printing for composite reconstruction of mandibular defects. Maxillofac Plast Reconstr Surg. (2022) 44:31. doi: 10.1186/s40902-022-00361-7, PMID: 36195777 PMC9532487

[115] Kumar BP Venkatesh V Kumar KAJ Yadav BY Mohan SR. Mandibular reconstruction: overview. J Maxillofacial Oral Surg. (2016) 15:425–41. doi: 10.1007/s12663-015-0766-5, PMID: 27833334 PMC5083680

[116] Cool S. Poly (3-hydroxybutyrate-co-3-hydroxyvalerate) composite biomaterials for bone tissue regeneration: in vitro performance assessed by osteoblast proliferation, osteoclast adhesion and resorption, and macrophage proinflammatory response. J Biomed Materials Res Part A Official J Society Biomat Japanese Society Biomat Australian Society Biomat Korean Society Biomat. (2007) 82:599–610. doi: 10.1002/jbm.a.31174 17315229

[117] Gentile P Chiono V Carmagnola I Hatton P. An overview of poly(lactic-co-glycolic) acid (PLGA)-based biomaterials for bone tissue engineering. Int J Mol Sci. (2014) 15:3640–59. doi: 10.3390/ijms15033640, PMID: 24590126 PMC3975359

[118] Gunatillake PA Adhikari R Gadegaard N. Biodegradable synthetic polymers for tissue engineering. Eur Cell Mater. (2003) 5:1–16. doi: 10.22203/eCM.v005a01 14562275

[119] Wang T Yang X Qi X Jiang C. Osteoinduction and proliferation of bone-marrow stromal cells in three-dimensional poly (ε-caprolactone)/hydroxyapatite/collagen scaffolds. J Transl Med. (2015) 13:1–11. doi: 10.1186/s12967-015-0499-8, PMID: 25952675 PMC4429830

[120] Matsubara T Suardita K Ishii M Sugiyama M Igarashi A Oda R. Alveolar bone marrow as a cell source for regenerative medicine: differences between alveolar and iliac bone marrow stromal cells. J Bone Miner Res. (2005) 20:399–409. doi: 10.1359/JBMR.041117 15746984

[121] Chung IH Yamaza T Zhao H Choung PH Shi S Chai Y. Stem cell property of postmigratory cranial neural crest cells and their utility in alveolar bone regeneration and tooth development. Stem Cells. (2009) 27:866–77. doi: 10.1002/stem.2, PMID: 19350689 PMC2896558

[122] Lin Z Fateh A Salem DM Intini G. Periosteum: biology and applications in craniofacial bone regeneration. J Dent Res. (2014) 93:109–16. doi: 10.1177/0022034513506445, PMID: 24088412 PMC3895334

[123] Shakoori P Zhang Q Le AD. Applications of mesenchymal stem cells in Oral and craniofacial regeneration. Oral Maxillofac Surg Clin North Am. (2017) 29:19–25. doi: 10.1016/j.coms.2016.08.009 27890225

[124] Tanikawa DYS. Deciduous dental pulp stem cells for maxillary alveolar reconstruction in cleft lip and palate patients. Stem Cells Int. (2020) 2020:6234167. doi: 10.1155/2020/6234167 32256610 PMC7091546

[125] Dental Stem Cells and Bone Tissue Engineering (CELSORDINO) (CELSORDINO). Central Hospital. France: Nancy (2017).

[126] Abdal‐Wahab M Abdel Ghaffar KA Ezzatt OM Hassan AAA El Ansary MMS Gamal AY. Regenerative potential of cultured gingival fibroblast- mesenchymal stem cells in treatment of periodontitis. Egypt: Ain Shams University (2018).

[127] Gungor-Ozkerim PS Inci I Zhang YS Khademhosseini A Dokmeci MR. Bioinks for 3D bioprinting: an overview. Biomater Sci. (2018) 6:915–46. doi: 10.1039/C7BM00765E, PMID: 29492503 PMC6439477

[128] Visscher DO Farré-Guasch E Helder MN Gibbs S Forouzanfar T van Zuijlen PP. Advances in bioprinting technologies for craniofacial reconstruction. Trends Biotechnol. (2016) 34:700–10. doi: 10.1016/j.tibtech.2016.04.001, PMID: 27113634

[129] Liu J Zhou Z Zhang M Song F Feng C Liu H. Simple and robust 3D bioprinting of full-thickness human skin tissue. Bioengineered. (2022) 13:10087–97. doi: 10.1080/21655979.2022.2063651, PMID: 35412953 PMC9161989

[130] Li M Sun L Liu Z Shen Z Cao Y Han L. 3D bioprinting of heterogeneous tissue-engineered skin containing human dermal fibroblasts and keratinocytes. Biomater Sci. (2023) 11:2461–77. doi: 10.1039/D2BM02092K, PMID: 36762551

[131] Kang H-W Lee SJ Ko IK Kengla C Yoo JJ Atala A. A 3D bioprinting system to produce human-scale tissue constructs with structural integrity. Nat Biotechnol. (2016) 34:312–9. doi: 10.1038/nbt.3413, PMID: 26878319

[132] Olson JL Atala A Yoo JJ. Tissue engineering: current strategies and future directions. Chonnam Med J. (2011) 47:1–13. doi: 10.4068/cmj.2011.47.1.1, PMID: 22111050 PMC3214857

[133] Partners AA. 3D tissue engineered bone equivalent for treatment of traumatic bone defects (3-D-TEBE). US: A.A. Partners, LLC (2017).

[134] Salah M Tayebi L Moharamzadeh K Naini FB. Three-dimensional bio-printing and bone tissue engineering: technical innovations and potential applications in maxillofacial reconstructive surgery. Maxillofac Plast Reconstr Surg. (2020) 42:18. doi: 10.1186/s40902-020-00263-6, PMID: 32548078 PMC7270214

[135] Wu C-C Lin PY Chew KY Kuo YR. Free tissue transfers in head and neck reconstruction: complications, outcomes and strategies for management of flap failure: analysis of 2019 flaps in single institute. Microsurgery. (2014) 34:339–44. doi: 10.1002/micr.22212, PMID: 24318866

[136] Ling XF Peng X Samman N. Donor-site morbidity of free fibula and DCIA flaps. J Oral Maxillofac Surg. (2013) 71:1604–12. doi: 10.1016/j.joms.2013.03.006, PMID: 23810616

[137] Kohlert S Quimby A Saman M Ducic Y. Postoperative free-flap monitoring techniques. Perspect Plas Surg. (2019) 33:13–016. doi: 10.1055/s-0039-1677880, PMID: 30863207 PMC6408240

[138] Chao AH Lamp S. Current approaches to free flap monitoring. Plas Aesthetic Nurs. (2014) 34:52–6. doi: 10.1097/PSN.0000000000000037 24887341

[139] Van Genechten M Rahmel B Batstone MD. Red or white? Use of high colour-rendering index, light-emitting diodes in monitoring of free flaps of the head and neck. Br J Oral Maxillofac Surg. (2015) 53:765–6. doi: 10.1016/j.bjoms.2015.04.020 25981628

[140] Kauke M Panayi AC Tchiloemba B Diehm YF Haug V Kollar B. Face transplantation in a black patient—racial considerations and early outcomes. N Engl J Med. (2021) 384:1075–6. doi: 10.1056/NEJMc2033961, PMID: 33730460 PMC8182672

[141] Knoedler S Hoch CC Huelsboemer L Knoedler L Stögner VA Pomahac B. Postoperative free flap monitoring in reconstructive surgery-man or machine? Front Surg. (2023) 10:1130566. doi: 10.3389/fsurg.2023.1130566, PMID: 36911625 PMC9992807

[142] SchöN R Schramm A Gellrich NC Maier W Jürgen D Schmelzeisen R. Color duplex sonography for the monitoring of vascularized free bone flaps. Otolaryngol Head Neck Surg. (2003) 129:71–6. doi: 10.1016/S0194-59980300486-8, PMID: 12869920

[143] Vakharia KT Henstrom D Lindsay R Cunnane MB Cheney M Hadlock T. Color doppler ultrasound: effective monitoring of the buried free flap in facial reanimation. Otolaryngol Head and Neck Surgery. (2012) 146:372–6. doi: 10.1177/0194599811427377 22261491

[144] Kempton SJ Poore SO Chen JT Afifi AM. Free flap monitoring using an implantable anastomotic venous flow coupler: analysis of 119 consecutive abdominal-based free flaps for breast reconstruction. Microsurgery. (2015) 35:337–44. doi: 10.1002/micr.22341, PMID: 25333860

[145] Han Z-F Guo LL Liu LB Li Q Zhou J Wei AZ. A comparison of the cook-Swartz doppler with conventional clinical methods for free flap monitoring: a systematic review and a meta-analysis. Int J Surg. (2016) 32:109–15. doi: 10.1016/j.ijsu.2016.06.034, PMID: 27353849

[146] Lindelauf AAMA Saelmans AG van Kuijk SMJ van der Hulst RRWJ Schols RM. Near-infrared spectroscopy (NIRS) versus hyperspectral imaging (HSI) to detect flap failure in reconstructive surgery: a systematic review. Life. (2022) 12:65. doi: 10.3390/life12010065, PMID: 35054458 PMC8778121

[147] Hallock GG. Acoustic doppler sonography, color duplex ultrasound, and laser doppler flowmetry as tools for successful autologous breast reconstruction. Clin Plast Surg. (2011) 38:203–11. doi: 10.1016/j.cps.2011.03.001, PMID: 21620145

[148] Monitoring of Tissue Transfer Flaps by Modulated Imaging (MI) Spectroscopy. The University of California Irvine. California, United States: Orange (2021).

[149] Monitoring of the Bone Free Flaps With Microdialysis (MTM2018). Centre Hospitalier Universitaire. France: Amiens (2023).

[150] Monitoring of Bone Free Flaps With Microdialysis (MTM). Amiens university hospital. Picardie, France: Amiens (2018).

[151] Chae MP Rozen WM Whitaker IS Chubb D Grinsell D Ashton MW. Current evidence for postoperative monitoring of microvascular free flaps: a systematic review. Ann Plast Surg. (2015) 74:621–32. doi: 10.1097/SAP.0b013e3181f8cb32, PMID: 23038130

[152] Laure B. Microdialysis: Experience in postoperative monitoring of 30 free flaps. Annales de Chirurgie plastique et Esthétique. Boston Ma: Little, Brown And Company (2008). 10.1016/j.anplas.2008.05.008 19042067

[153] Mourouzis C Anand R Bowden JR Brennan PA. Microdialysis: use in the assessment of a buried bone-only fibular free flap. Plast Reconstr Surg. (2007) 120:1363–6. doi: 10.1097/01.prs.0000279555.75241.4c, PMID: 17898613

[154] Dakpé S Colin E Bettoni J Davrou J Diouf M Devauchelle B. Intraosseous microdialysis for bone free flap monitoring in head and neck reconstructive surgery: a prospective pilot study. Microsurgery. (2020) 40:315–23. doi: 10.1002/micr.30529, PMID: 31638286 PMC7155115

[155] Wang Y Qu X Jiang J Sun J Zhang C He Y. Aesthetical and accuracy outcomes of reconstruction of maxillary defect by 3D virtual surgical planning. Front Oncol. (2021) 11:8946. doi: 10.3389/fonc.2021.718946 PMC8560731 34737946

[156] Chan T Long C Wang E Prisman E. The state of virtual surgical planning in maxillary reconstruction: a systematic review. Oral Oncol. (2022) 133:106058. doi: 10.1016/j.oraloncology.2022.106058, PMID: 35952582

[157] Bassani S Eccher A Molteni G. Harnessing the power of AI: Revolutionizing free flaps monitoring in head and neck tumor treatment. Critical reviews™ in oncogenesis. Boston Ma: Little, Brown And Company (2022). 10.1615/CritRevOncog.2023049158 37968990

[158] Huang RW Tsai TY Hsieh YH Hsu CC Chen SH Lee CH. Reliability of postoperative free flap monitoring with a novel prediction model based on supervised machine learning. Plast Reconstr Surg. (2023). doi: 10.1097/PRS.0000000000010307, PMID: 36790782

[159] Kiranantawat K Sitpahul N Taeprasartsit P Constantinides J Kruavit A Srimuninnimit V. The first smartphone application for microsurgery monitoring: SilpaRamanitor. Plast Reconstr Surg. (2014) 134:130–9. doi: 10.1097/PRS.0000000000000276, PMID: 25028822

[160] Hsu S-Y Chen LW Huang RW Tsai TY Hung SY Cheong DC. Quantization of extraoral free flap monitoring for venous congestion with deep learning integrated iOS applications on smartphones -- a diagnostic study. Int J Surg. (2023) 109:1584–93. doi: 10.1097/JS9.0000000000000391, PMID: 37055021 PMC10389505

[161] Provenzano D Chandawarkar A Caterson E. Novel smartphone based free flap monitoring tool using machine learning. bioRxiv. (2022) 7. doi: 10.1097/01.GOX.0000558434.13952.7c

[162] Crawley MB Sweeny L Ravipati P Heffelfinger R Krein H Luginbuhl A. Factors associated with free flap failures in head and neck reconstruction. Otolaryngol Head Neck Surg. (2019) 161:598–604. doi: 10.1177/0194599819860809 31382816

[163] van Gemert JTM Abbink JH van Es RJJ Rosenberg AJWP Koole R van Cann EM. Early and late complications in the reconstructed mandible with free fibula flaps. J Surg Oncol. (2018) 117:773–80. doi: 10.1002/jso.24976, PMID: 29448299 PMC5901040

[164] Sweeny L Topf M Wax MK Rosenthal EL Greene BJ Heffelfinger R. Shift in the timing of microvascular free tissue transfer failures in head and neck reconstruction. Laryngoscope. (2020) 130:347–53. doi: 10.1002/lary.28177, PMID: 31287566

